# A simple approach to hybrid inorganic–organic step-growth hydrogels with scalable control of physicochemical properties and biodegradability†
†Electronic supplementary information (ESI) available: Experimental details and characterization data as mentioned in the text. See DOI: 10.1039/c4py01789g



**DOI:** 10.1039/c4py01789g

**Published:** 2015-02-11

**Authors:** F. Alves, I. Nischang

**Affiliations:** a Institute of Polymer Chemistry , Johannes Kepler University Linz , A-4060 Leonding , Austria . Email: ivo.nischang@jku.at

## Abstract

We prepared new and scalable, hybrid inorganic–organic step-growth hydrogels with polyhedral oligomeric silsesquioxane (POSS) network knot construction elements and hydrolytically degradable poly(ethylene glycol) (PEG) di-ester macromonomers by *in situ* radical-mediated thiol–ene photopolymerization. The physicochemical properties of the gels are fine-tailored over orders of magnitude including functionalization of their interior, a hierarchical gel structure, and biodegradability.

## Abstract 2

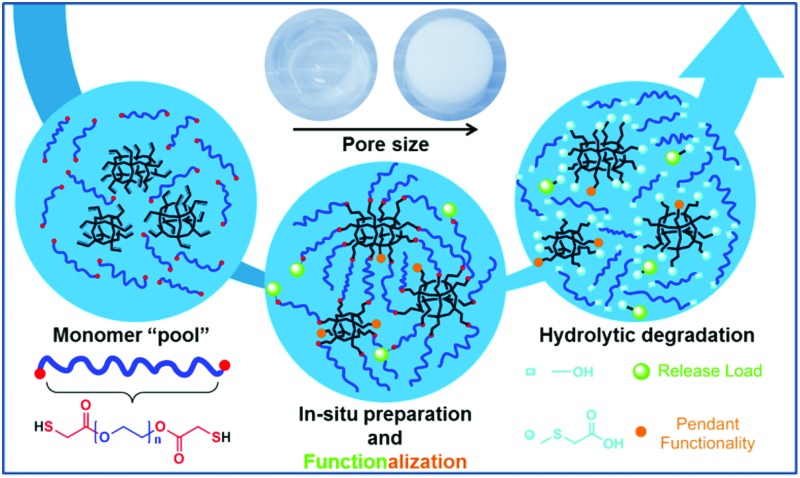
We prepared new and scalable, hybrid inorganic–organic step-growth hydrogels with polyhedral oligomeric silsesquioxane (POSS) network knot construction elements and hydrolytically degradable poly(ethylene glycol) (PEG) di-ester macromonomers by *in situ* radical-mediated thiol–ene photopolymerization.

Chemically cross-linked materials with the ability to sorb and retain water, *i.e.* hydrogels, are of outstanding importance for biomedical purposes, such as tissue engineering and drug delivery.^1^ Their water storage ability alongside their soft, tissue-like properties allows them to mimic several biological environments.

In the last decade, the search for rationally controlling the derivation of hydrogels with improved performance led to a shift in the preparation strategy from free radical (co)polymerization to step-growth approaches.^2^ Though a wide set of chemistries have been suggested to this end, PEG is used in majority as one key starting precursor due to its well-recognized biocompatibility.^3^

The exploitation of novel key precursors to derive hydrogels of nanoscale homogeneity with enhanced mechanical strength and tailored biodegradability together with straightforward introduction of suitable functionality is an area of profound relevance. This is stimulated by the high demand of application-specific versatility.

Polyhedral oligomeric silsesquioxanes (POSS) with a multiplicity of functional vertices are cage-like, cytocompatible nanostructures that have aroused attention for the creation of hybrid inorganic–organic materials of outstanding outreach. This is recognized for high-end applications such as chromatography, extraction, nanomedicine, *etc.* ^4^ Poly(*N*-isopropylacrylamide) (PNIPAAm) based hydrogels incorporating POSS have been reported to give the tailorability of thermoresponse.^5^ However, PNIPAAm based materials have revealed very limited suitability for biomedical purposes.^6^

We hypothesize and show that the introduction of POSS as network knots within hydrogels may lead to an improved mechanical strength giving the small, though rigid, inorganic silica framework. Furthermore, the multiplicity of functionality may enable a desirable cross-link density with remaining (functionalized) vertices being left at appropriate stoichiometry for further fine-tailoring of the gels’ internal structure. To the best of our knowledge, the utilization of POSS precursors for the derivation of molecularly defined step-growth hydrogels with potential use in biomedical-related areas has not yet been realized. This is surprising since vinyl functional POSS undergoes an exclusive radical mediated step-growth reaction with thiols.^7^ Herein, we introduce the facile preparation and characterization of a novel set of hybrid inorganic–organic hydrogels that utilize vinylPOSS and homotelechelic thiol PEG di-ester macromonomers (ESI†) as precursors for the creation of (functional) 3D networks by step-growth thiol–ene photopolymerization.

Illustration of the materials’ principal nanoscale internal structure alongside resulting products of hydrolytic degradation can be found in Scheme 1.

**Scheme 1:**
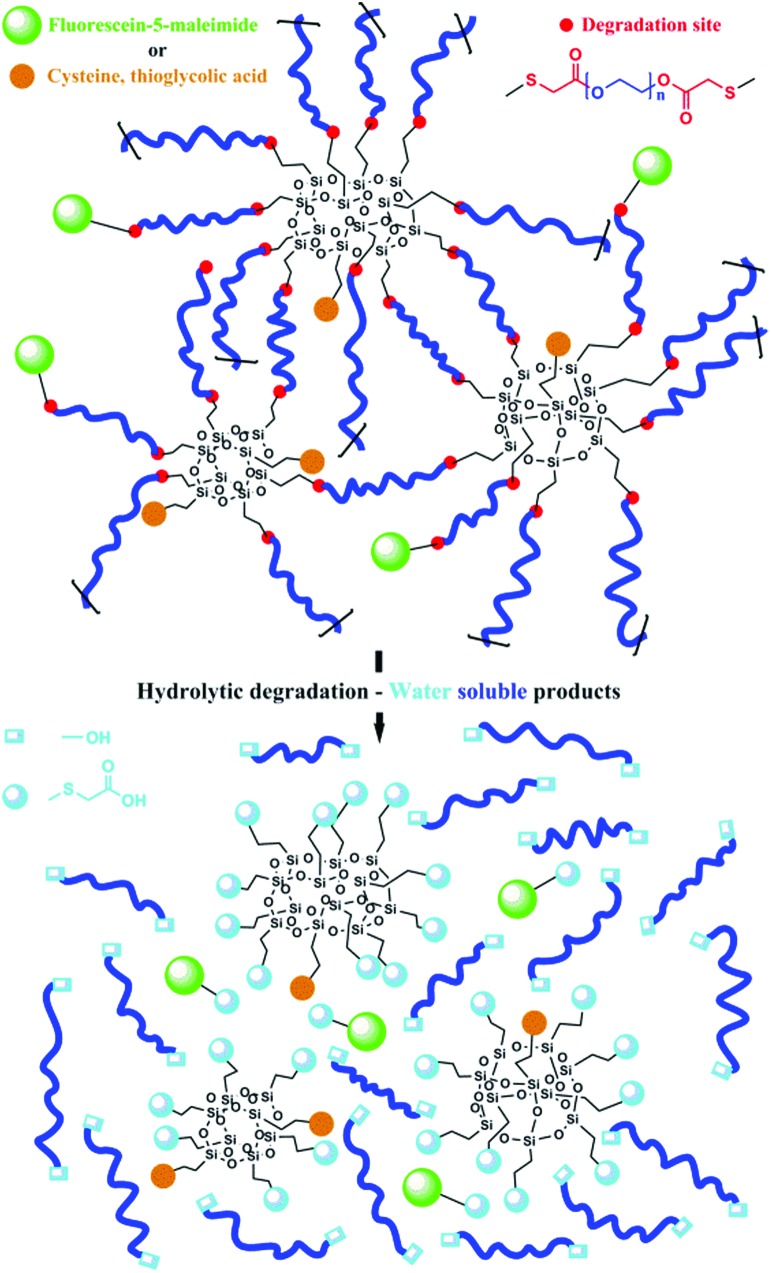
Example of the hybrid inorganic–organic nanoscale gel structures prepared on the basis of vinylPOSS ((RSiO_3/2_)_*n*_ shown for *n* = 8, 10, and 12) and homotelechelic thiol PEG di-ester macromonomers together with the resulting products upon hydrolytic degradation.

The homogeneous precursor solutions used to prepare the hybrid hydrogels are detailed in Table 1 alongside determined gel fractions.

**Table 1:** Overview of prepared hybrid hydrogels

Gel	*M*_n_ PEG^*a*^ (g mol^–1^)	Thiol : vinyl^*b*^ (–)	wt% Dodecanol^*c*^	% Gel fraction^*d*^
**1**	748.24	1 : 1	—	92.7 (±0.9)
**2**	1148.24	1 : 1	—	89.6 (±2.4)
**3**	2198.24	1 : 1	—	82.7 (±0.3)
**4**	6148.24	1 : 1	—	71.5 (±2.6)
**5**	1148.24	1 : 1.5	—	92.5 (±1.2)
**6**	1148.24	1.5 : 1	—	75.1 (±2.6)
**7**	1148.24	2 : 1	—	57.0 (±1.3)
**8**	1148.24	1 : 1	30	89.7 (±1.0)
**9**	1148.24	1 : 1	50	88.1 (±0.9)
**10**	1148.24	1 : 1	60	88.9 (±0.3)

^*a*^ *M*_n_ of the PEG macromonomer calculated on the basis of the *M*_n_ of the pristine PEG (provided by the manufacturer) plus the molecular weight of two units of thioglycolic acid minus two units of water.

^*b*^Total wt% of monomers to the solvent DMF was 20 : 80. The polymerization mixtures additionally contained 1 wt% DMPA (with respect to thiol).

^*c*^Specific portions of DMF were replaced by the co-solvent dodecanol.

^*d*^Gel fraction was determined as the ratio of the dry weight of the gel divided by the overall weight of monomers used for preparation. Values in brackets represent standard deviation from three independent gel fraction determinations.

Fig. 1a clearly shows an increased volume at an increased PEG macromonomer chain length, a clear indication of the hydrogels larger free volume that contains water. The swelling ratio increased from 180% for Gel **1** to 2080% for Gel **4** (Fig. 2a). This increase in water uptake shows proportionality to the increase in the PEG macromonomer chain length for Gels **1–3**, indicating the attainment of a step-growth homogeneous network (Fig. S1, ESI†). A lower gel fraction for Gel **4** explains increased water uptake. The Fourier transform infrared (FTIR) spectroscopy (Fig. S2a, ESI†) and Raman spectra (Fig. S2b, ESI†) clearly show similar features of Gels **1–4** with a progressively reduced intensity of the C=O str band (1730 cm^–1^).

**Fig. 1:**
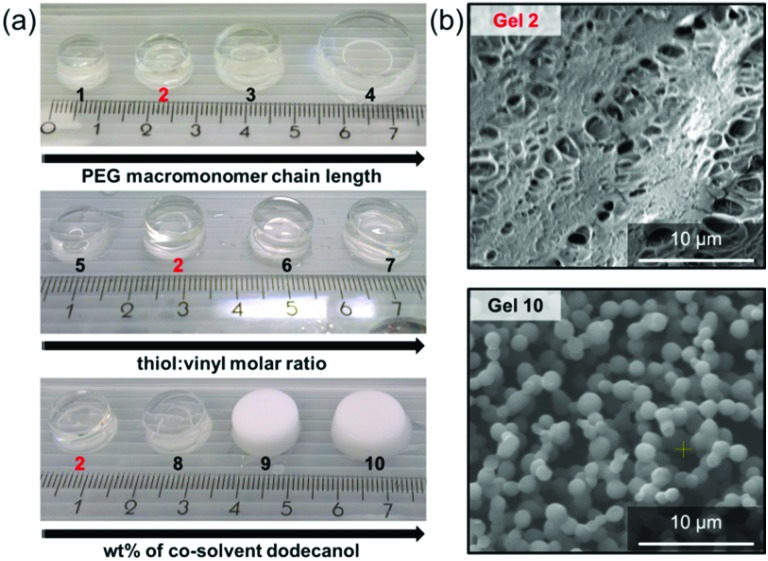
(a) Photographs of the water-containing hydrogels prepared according to Table 1. All materials were prepared by molding within 4 mL glass vials. Thus, the volume variations observed are due to inherently different water uptakes of the gels. (b) Cryo-SEM of Gels **2** and **10**.

**Fig. 2:**
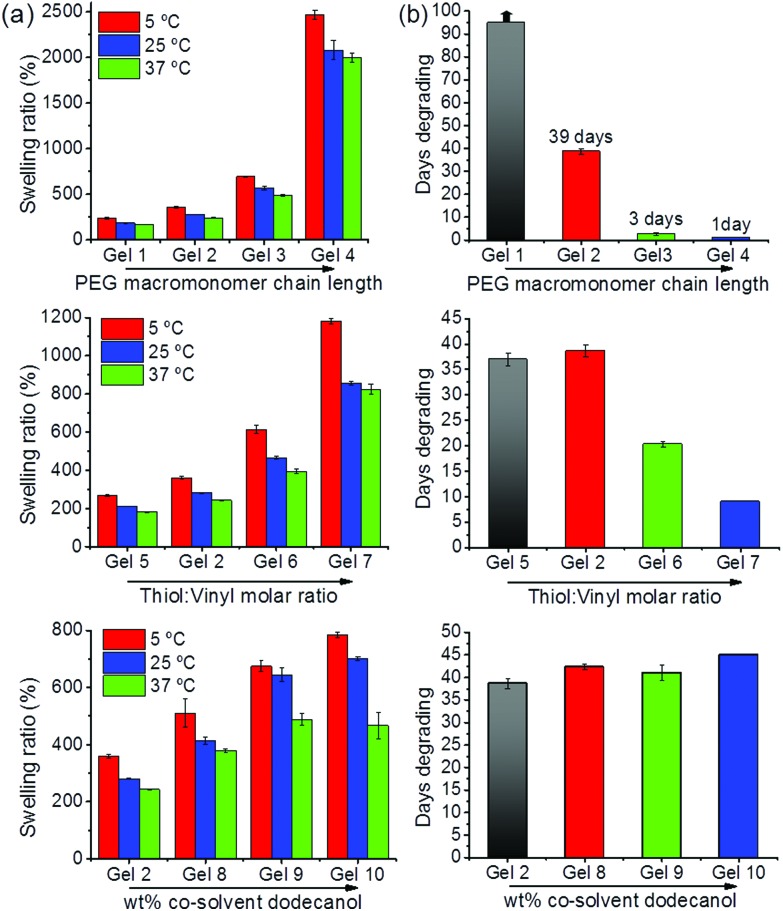
(a) Deionized water swelling ratios at *T* = 5 °C (red bars), *T* = 25 °C (blue bars), and *T* = 37 °C (green bars) for hydrogels prepared according to Table 1 at increased PEG macromonomer chain lengths (Gels **1–4**), increased thiol : vinyl molar ratios (Gels **2**, and **5–7**), and increased wt% of the co-solvent dodecanol used for gel preparation (Gel **2**, and Gels **8–10**). (b) Time taken to complete degradation of the hydrogels prepared according to Table 1 and shown in the left row immersed in 15 mL 0.1 M PBS at pH = 7.4 and *T* = 37 °C to mimic physiological conditions.

Choice of varying stoichiometric ratios of vinyl : thiol (Gels **5**, **6**, and **7**) with one example PEG macromonomer (*M*_n_ = 1148.24 g mol^–1^) shows that an excess of thiol functionality (Gels **6** and **7**) resulted in gels that show more pronounced volume increase at reduced gel fractions (Table 1, Fig. 1a). The complete disappearance of the vinyl band together with a signal for the thiol band in Gels **6** and **7**, disappearance of both in Gel **2**, and existence of the vinyl band in Gel **5**, indicate the step-growth mechanism of gel formation at varying cross-linking density (Fig. S3a, ESI†). Furthermore, Gel **4** clearly shows a much larger volume in the swollen state than Gels **6** and **7**. Their gel fractions are equal or smaller than Gel **4**. This shows that the larger length of the PEG macromonomer in the first series of experiments is a major cause of the progressive volume increase. The inorganic amorphous silica residues (Fig. S4, ESI†) left from the thermogravimetric analysis (TGA) of dried Gels **6** and **7** indicate higher than expected POSS content based on stoichiometric choice (Table S1, ESI†). At reduced gel fractions part of the PEG macromonomers used in excess are not incorporated into the resulting networks. These results also reveal that higher cross-linking degrees (at a constant PEG macromonomer chain length) are attained at equimolar concentrations of vinyl to thiol groups (Gel **2**) and at an excess of vinyl groups (Gel **5**). Gels **2** and **5** have highest gel fractions (Table 1).

In the third series of experiments (Gels **8–10**), the PEG macromonomer chain length remained constant (*M*_n_ = 1148.24 g mol^–1^) at equimolar concentration of thiol and vinyl functional groups and we stimulated polymerization-induced phase separation by employing the co-solvent dodecanol. This resulted in gels with an opaque appearance (Gels **9** and **10**), larger volume, and larger water uptake (Fig. 1a and Fig. 2a). Gel **10** shows more than twice the swelling ratio of Gel **2** (Fig. 2a). Nevertheless, gel fractions of Gels **2** and **8–10** were found similar (Table 1) together with very similar ceramic yields from TGA (Table S1, ESI†). The results become further underpinned by the gels’ identical Raman spectra (Fig. S3b, ESI†). Cryogenic scanning electron microscopy (cryo-SEM) images show micrometer-sized pores formed by particle-shaped, 3D interconnected structural features for Gel **10** in contrast to a much denser structure found for Gel **2** (Fig. 1b). A hierarchical (porous) gel structure with large, micrometer-sized pores and the identical cross-linked gel material by itself are able to accommodate more water (Fig. 2b).

As also seen in Fig. 2a, the inherent water uptake of the hybrid hydrogels was found to be significantly dependent on temperature. In fact, when lowering the temperature from *T* = 25 °C to 5 °C, all gels absorbed an additional 20 wt% water. By raising the temperature from *T* = 25 °C to 37 °C all gels expelled about 10 wt% water. This temperature-dependent water uptake was considerably more pronounced when compared to that reported in the literature for PEG-based amphiphilic hydrogels.^8^ This situation may be associated with the more homogeneous amphiphilic step-growth network created. Such dependence may further be tuned and exploited for delivery and actuation purposes.

As can be seen in Fig. 3, compressive stress–strain curves reflect a wide variety of shapes and slopes revealing the gels’ substantially different mechanical properties. An increase of the PEG macromonomer chain length exemplified, *e.g.* by Gels **2** and **4** led to progressively softer materials. For example, the compressive moduli decreased from 465.5(±63.0) kPa (Gel **2**) to 21.8(±4.6) kPa (Gel **4**), more than an order of magnitude. Tuning the vinyl : thiol stoichiometric ratio led to hydrogels of softer nature at an excess of thiol, *e.g.* by comparing Gels **2** and **6**, whose compressive moduli decreased from 465.5(±63.0) kPa (Gel **2**) to 133.2(±24.8) kPa (Gel **6**), respectively. Overall, these determined values are favorable to those recently reported for PEG-based hydrogels additionally reinforced with polydimethylsiloxane.^9^ Creating large pores in the gels lead to softening, but to a much enhanced ability to stand deformation without rupture. For example, Gel **10** exhibited a compressive modulus of only 3(±0.3) kPa, respectively, but it could stand deformations as large as 90% compressive strain without apparent rupture. Here, Gel **10** revealed a compressive stress of 1031.6(±40.9) kPa. We believe that the hierarchical gel structure observed in Fig. 1b provides a better relaxation of stresses. Polymerization induced phase separation may, therefore, be a simple approach to improve and tailor the mechanical properties of each of the here presented hydrogels without impairing their chemical constitution.

**Fig. 3:**
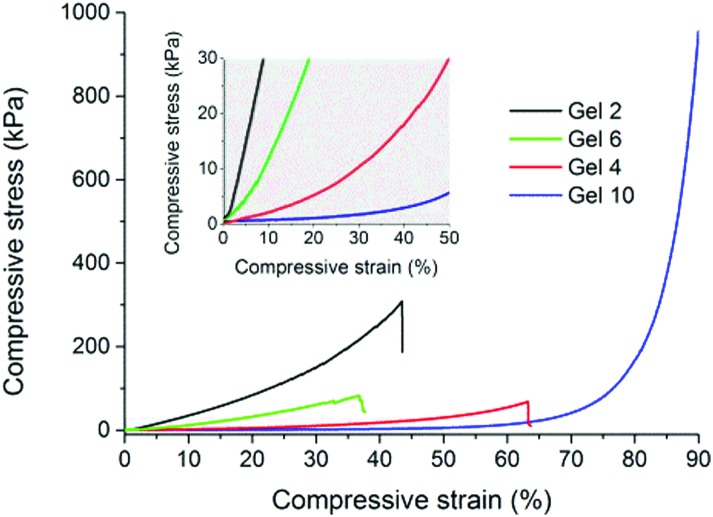
Compressive stress–strain curves obtained through uniaxial compression of selected gels prepared according to Table 1. The inset shows an enlarged view of the initial slopes observed in the grey-shaded area. A detailed summary of mechanical data extracted from such curves is reported in Table S2 in the ESI.†

Choice of stoichiometry allows for functionalization of the internal structure of gels. In order to demonstrate the feasibility of remaining vinyl or thiol groups to undergo further functionalization, we modified them with thioglycolic acid and cysteine (Gel **5**), and the dye fluorescein-5-maleimide (Gel **7**). The addition of cysteine and thioglycolic acid to Gel **5** was confirmed by Raman spectroscopy through the significant reduction of the vinyl band at 1600 cm^–1^ (Fig. S5a, ESI†). Adding cysteine also appeared to enhance water uptake by 40% due to the pendant zwitterionic functionality in the hydrogel. The addition of the fluorescein-5-maleimide to Gel **7** in the absence of an initiator was readily confirmed by irradiating the resulting hydrogel piece with 365 nm wavelength UV-light. Here, it emitted green fluorescent light (see inset in Fig. S5b, ESI†). Furthermore, the Raman spectrum showed a significant reduction of the thiol band at 2570 cm^–1^ in comparison with the spectrum of the pristine material (Fig. S5b, ESI†).

While gels possessed reasonable stability in de-ionized water without significant notion of short-term degradation, the ester moieties inherent to the network showed tendency for degradation under close to physiological conditions in phosphate buffer solution (PBS) at *T* = 37 °C and a pH of 7.4. Hydrolytic degradation of the ester moieties results in PEG with defined sizes and new functional POSS entities. Released POSS contains mainly carboxylic acid functionalities and other synthetically determined functionalities (Scheme 1). Thus, the inherent existence of ester moieties in these networks leads to biodegradability with water soluble and non-toxic degradation products.^10^

Fig. 2b shows the wide range of adjustable degradation under near physiological conditions with Gel **1** revealing the slowest degradation. Its structural integrity remained over significant time scales (Fig. S6, ESI†). On the other hand, Gel **2** required about 39 days to disappear in solution and Gels **3** and **4** degraded within 3 days and 1 day, respectively (Fig. 2b). These results are associated with the gels’ reduced cross-link density and consequently reduced amount of degradable ester moieties per unit of volume.

Through adjustment of the vinyl : thiol molar ratio used in preparation of the gels (Gels **5–7**), degradation could also be tuned. Gels **6** and **7** take about 20 and 9 days to degrade, considerably faster than Gel **2** with Gel **5** taking comparable time as Gel **2** to completely degrade. Gel **5** accommodates lower amounts of water than Gel **2** (Fig. 2a) due to the existence of residual vinyl functionalities and poorer solvation of the direct vicinity of the POSS cage. By functionalization of the pendant vinyls in Gel **5** either with cysteine or thioglycolic acid, degradation is accelerated significantly (Fig. S7, ESI†). Gel **7** functionalized with fluorescein-5-maleimide was placed in PBS and the vial irradiated with 365 nm UV light. Shortly thereafter, Gel **7** started releasing fluorescein, visually indicative of the ester moiety degradation that immobilizes it in the hydrogel network (Scheme 1, Fig. S8, ESI†). After three days, the entire solution was pronouncedly fluorescent. Placing the gel in de-ionized water showed the remaining, highly fluorescent pieces of the hydrogel.

No considerable difference among degradation times was observed for Gels **2**, and **8–10** (Fig. 2b) expected from the same macromonomer chain length, chemical constitution but a hierarchical gel structure (Fig. 1b).

In conclusion, we have clearly demonstrated how a new class of hybrid, step-growth hydrogels with enhanced and widely varying physicochemical characteristics can be prepared by means of a straightforward thiol–ene photopolymerization in standard glassware. The related physicochemical properties of the materials are tunable over orders of magnitude. This is enabled by a rational choice of here presented design criteria, *i.e.* (i) PEG-macromonomer chain length, (ii) stoichiometry of functionality used for preparation, and (iii) a fine-tailored internal (templated) porous structure through polymerization-induced phase separation. The last aspect can be realized by an identical chemical nature of materials at modulated physical characteristics. Controlled introduction of internal functionality further tailors the gels related properties and their sensitivity to degradation. The facile ability to manipulate the gels’ functionality and porous structure alongside their inherent temperature-dependent water uptake and tunable biodegradability turns these versatile set of materials as potential candidates for biomedical-related applications. For applications such as tissue engineering, controlled porous properties, functionality, and degradation are all aspects that are deemed important and have been demonstrated here. Though demonstrated for PEG macromonomers, the presented principal strategy is not limited to it, rather than to any biocompatible macromolecular linker possessing at least two pendant thiol groups.

This work was supported by the Austrian Science Fund (FWF) under project number [P24557-N19]. The authors acknowledge Karin Whitmore (University Service Center for Transmission Electron Microscopy, Vienna University of Technology, Vienna, Austria) for support with the cryo-SEM measurements and Andreas Gösweiner (Transfercenter für Kunststofftechnik GmbH (TCKT), Wels, Austria) for support with the mechanical measurements. We kindly acknowledge the Research Center for Non Destructive Testing (RECENDT) GmbH for allowing us to use their portable Raman spectrometer.
