# Supplementary material for: A simple approach to hybrid inorganic–organic step-growth hydrogels with scalable control of physicochemical properties and biodegradability
Source: Polym Chem. 2015 Feb 11;6(12):2183–7. doi: 10.1039/c4py01789g (PMC4360384; doi:10.1039/c4py01789g)
Supplement: Supplementary file 1 [file PY-006-C4PY01789G-s001.pdf]

Electronic Supplementary Material (ESI) for Polymer Chemistry.
This journal is © The Royal Society of Chemistry 2015

Supplementary Material (ESI)

Simple Approach to Hybrid Inorganic-Organic Step-Growth Hydrogels with Scalable Control of Physicochemical Properties and Biodegradability

Filipa Alves and Ivo Nischang*

Institute of Polymer Chemistry, Johannes Kepler University Linz, Welser Str. 42, 4060 Leonding (Austria)

*E-mail: ivo.nischang@jku.at, tel.: + 43 (0) 732671547-66

Experimental Section

Equipment. The UV-light triggered reactions were performed in a Rayonet Chamber Reactor with UV-lamps having their maximum intensity located at a wavelength of 253 nm. We used varying illumination times as detailed (vide infra). The temperature in the reactor was stable at T = 22 ºC.

Solution ^{1}H and ^{13}C NMR spectra were recorded on a Bruker 300 MHz spectrometer with tetramethylsilane [$(CH_3)_4Si$; TMS] as the reference.

Fourier transform infrared (FTIR) spectra of respective materials (precursors and gels) were acquired with a Perkin Elmer Spectrum 100 ATR-FTIR spectrometer while Raman spectra were obtained with a portable i-Raman Plus, B&WTek spectrometer equipped with a 785 nm laser. A laser intensity of 100 % was used in all experiments.

Cryogenic scanning electron microscopy (cryo-SEM) was performed on a Quanta 200 3D DualBeam-FIB equipped with a cryo-transfer stage and a cryo-preparation chamber. Water equilibrated hydrogel pieces were rapidly frozen by immersing them in liquid nitrogen under

vacuum and quickly transferred into the cryo-preparation chamber at a temperature of T = -90 ºC. Inside this chamber, the frozen samples were fractured to expose a virgin surface and ice was allowed to sublimate for 1 hour in vacuum before sputtering a thin layer of gold and subsequent imaging.

Thermal gravimetric analysis (TGA) of the dried hydrogels were measured in a TA Q5000 instrument with heating ramps of 10 ºC min^{-1} from T = 50 ºC to 800 ºC under air atmosphere.

Compression tests of the hydrogel discs were performed in a Zwick/Roell Z0.5 mechanical tester using a load cell of 500 N and a compression velocity of 500 µm min^{-1}. The water-equilibrated hydrogel discs were objected to compression tests in an unconfined setting. Dependent upon water-uptake of the respective sample, the dimensions of the discs tested varied from approximately 5 to 6 mm in thickness and from 11 to 15 mm in diameter. The compression strain limit was set to 90%. The compressive moduli were determined from the slope of the initial near-linear part of the stress-strain curve until 10% strain.

Materials. Polyhedral oligomeric vinylsilsesquioxane (vinylPOSS) cage mixture $(CH_2CHSiO_{3/2})_n$ (n= 8, 10, 12) with a molecular weight of 633-950 g mol^{-1}, was purchased from Hybrid Plastics, Inc. (Hattiesburg, USA). Water was purified on a Milli-Q Reference water purification system from Millipore (Vienna, Austria). Dimethylformamide (DMF), deuterated chloroform ($CDCl_3$), chloroform ($CHCl_3$), and diethylether (DEE) were purchased from VWR (Vienna, Austria). Poly(ethylene glycol) (PEG) Ph. Eur. with a number average molecular weight (M_n) of 600, 1000, 2050, and 6000 g mol^{-1}, thioglycolic acid, the photoinitiator 2,2-dimethoxy-2-phenylacetophenone (DMPA), anhydrous benzene, and L-Cysteine were acquired from Sigma Aldrich (Vienna, Austria). Concentrated (95-98 %) sulphuric acid was purchased from J.T. Baker (Daventer, Holland). Fluorescein-5-maleimide was purchased from Tokyo Chemical Industry (TCI, Eschborn, Germany). All chemicals

were used as received. Standard 4 mL glass vials with a screw cap have been used as a mold for the in-situ photoinitiated hydrogel formation.

Preparation of Homotelechelic Thiol PEG Di-Esters (PEG Macromonomers). Thioglycolic acid, the respective PEG and concentrated sulphuric acid were dissolved in benzene and allowed to stir for 1 hour under nitrogen atmosphere. The solution was then heated to the refluxing temperature and allowed to react overnight. The molar equivalents (equiv.) of thioglycolic acid to that of hydroxyl functionality of PEG varied from 2.5 equiv. for the PEG with M_n = 600 and 1000 g mol^{-1} to 4 equiv. and 30 equiv. for M_n = 2050 and 6000 g mol^{-1}, respectively. Water from the esterification reaction formed an azeotrope with benzene and in this fashion was removed from the system with a Dean-Stark trap. After reaction, benzene was removed by vacuum distillation and the reaction product was subsequently taken up in chloroform. The PEG M_n = 748.24 g mol^{-1} macromonomer (which appeared as a transparent liquid) was washed twice with water in a separation funnel with the organic phase being concentrated thereafter by rotary evaporation. The other PEG macromonomers (which typically appeared as white solids) were precipitated twice from chloroform by addition of diethylether. All PEG macromonomers were then placed in a desiccator for drying and storage before use or further characterization. The above-mentioned procedure resulted in the following yields and NMR results of PEG macromonomers:

PEG M_n = 748.24 g mol^{-1}: 89% - ^{1}H NMR ($CDCl_3$, 300 MHz): δ_{ppm} 2.03 (t, 2H, J = 8.4 Hz), 3.29 (d, 4H, J = 8.2 Hz), 3.75 - 3.57 (m, 51H), 4.29 (t, 4H, J = 4.6 Hz), ^{13}CNMR ($CDCl_3$, 75 MHz): δ_{ppm} 26.44, 64.70, 68.88, 70.55, 170.90;

PEG M_n = 1148.24 g mol^{-1}: 62% - ^{1}H NMR ($CDCl_3$, 300 MHz): δ_{ppm} 2.00 (t, 2H, J = 8.3 Hz), 3.27 (d, 4H, J = 8.3 Hz,), 3.72 - 3.54 (m, 90H), 4.27 (t, 4H, J = 4.7 Hz), ^{13}CNMR ($CDCl_3$, 75 MHz): δ_{ppm} 26.44, 64.70, 68.88, 70.55, 170.90;

PEG M_n = 2198.24 g mol^{-1}: 65% - ^1H NMR ($CDCl_3$, 300 MHz): δ_{ppm} 2.04 (t, 2H, J = 8.2 Hz), 3.30 (d, 4H, J = 8.2 Hz), 3.77 - 3.50 (m, 181H), 4.30 (t, 4H, J = 4.6 Hz), ^{13}CNMR ($CDCl_3$, 75 MHz): δ_{ppm} 26.43, 64.69, 68.88, 70.54, 170.90;

PEG M_n = 6148.24 g mol^{-1}: 93% - ^1H NMR ($CDCl_3$, 300 MHz,): δ_{ppm} 2.00 (t, 2H, J = 8.3 Hz), 3.26 (d, 4H, J = 8.3 Hz), 3.75 - 3.42 (m, 481H), 4.26 (t, 4H, J = 4.6 Hz), ^{13}CNMR ($CDCl_3$, 75 MHz): δ_{ppm} 26.45, 64.71, 68.89, 70.54, 170.91.

Similar peak shifts, integration amplitudes as well as coupling constants were also reported by other authors working with related PEG macromonomers.[1]

Hydrogel Preparation. For hydrogel preparation, the vinylPOSS cage mixture and radical initiator (DMPA) (1 wt% with respect to PEG macromonomer) was first dissolved in DMF and then the PEG macromonomer was added.

In a first and second series of experiments, this procedure was followed with an equimolar concentration of functional groups while varying the PEG macromonomer chain length (Gels 1-4), and with a disparity of functional group concentration with one example PEG macromonomer chain length (M_n = 1148.24 g mol^{-1}) (Gels 5-7). Additionally, in a third series of experiments the PEG macromonomer chain length remained constant at equimolar concentration of thiol and vinyl functional groups and we applied the co-solvent dodecanol that was added after the dissolution of the gel precursor monomer mixture in respective amounts of DMF (Gels 8-10). The homogeneous liquid precursor solutions were transferred to 4 mL glass vials, and sealed with the cap. UV-initiated reactions were triggered by placing the vials in a Rayonet Chamber reactor. Following gel formation, the resulting disc-shaped materials were placed in glass vials with a frequent exchange of solvent DMF in order to remove any non-reacted monomers and initiator. Subsequently, DMF was gradually exchanged to deionized water, in which the hydrogels were allowed to equilibrate for one day under frequent water exchange before further characterization.

Hydrogel Functionalization. Hydrogel discs prepared with a stoichiometric disparity of functional groups, resulting in gels containing vinyl- or thiol-pendant moieties, underwent further functionalization. Vinyl-pendant hydrogels (Gel 5) were functionalized in a solution of 80:20 water/DMF (%, v/v) containing 5 % (w/v) of cysteine or thioglycolic acid with 1 wt% DMPA (with respect to the thiol). Reactions were performed in the UV reactor for one hour under stirring. The functionalized gels were purified by frequent exchange of a solution of 80:20 water and DMF (%, v/v) before gradual exchange to deionized water, followed by further characterization. An example gel containing thiol-pendant functionality (Gel 7) was functionalized with fluorescein-5-maleimide. Therefore, the DMF containing hydrogel was placed in 4 mL DMF containing 10 mg of the dye. The sealed reaction vessel was placed in an oil bath overnight at 60 ºC under stirring. Thereafter, the hydrogel was thoroughly washed with DMF to remove non-reacted species. Then, the solvent was exchanged to deionized water and the gel further characterized.

Determination of Gel Fractions and Water Uptake. The gel fraction is a useful estimate to judge on the efficiency of transformation of monomeric precursors to actually incorporated amount of these precursors in the final gel. Therefore, after formation of gels, washing, and subsequent exchange to water, they were dried, and the gel fraction calculated via the following equation:

$$Gel\ fraction = \frac{Weight_{gel,\ dry}}{Weight_{precursors}} \qquad \text{(eq. 1)}$$

For characterization of water content under equilibrium conditions, the weights of the water-containing hydrogel discs at room temperature were recorded after quickly wiping off the excess of water on their surfaces with moistened tissue. Thereafter, control samples were allowed to dry at room temperature, while others were placed either in a water bath at T = 37

ºC or in a cold room at T = 5 ºC for one day. Afterwards, the corresponding weights were determined as mentioned before. Finally, also these materials were allowed to dry at room temperature until their weight did not reduce any further. Percent swelling ratios were calculated according to the following equation.

$$Swelling\ ratio = \frac{Weight_{gel,\ wet} - Weight_{gel,\ dry}}{Weight_{gel,\ dry}} \times 100\ \% \qquad \text{(eq. 2)}$$

Hydrogel Degradation. To evaluate the tendency of gels to degrade, water-equilibrated hydrogel pieces with masses between 50 and 150 mg (depending on the varying swelling ratios) were placed in 15 mL 0.1 M phosphate buffer solution (PBS) with a pH of 7.4 and at T = 37 ºC (near physiological conditions). The buffer solution was exchanged weekly to remove degradation products. The tests were performed until complete degradation of the hydrogels was observed.

Additional Tables

Table S1: Comparison of theoretical and experimentally determined ceramic yields (SiO_2) from TGA analysis of all hydrogels.

Gel	[a]Ceramic SiO_2 yield from TGA analysis (wt%)	[b]Theoretically expected SiO_2 ceramic yield (wt%)
1	13.4	13.3
2	9.5	9.2
3	5.6	5.1
4	2.2	1.9
5	13.4	13.0
6	8.0	6.4
7	7.6	4.9
8	9.0	9.2
9	9.6	9.2
10	9.6	9.2

[a]Values determined from the end of TGA degradation curves at 800 °C.

[b]Values calculated based on the assumption that all POSS precursors are incorporated in the polymer in correct stoichiometric amounts and the $SiO_{3/2}$ contained in the respective

hydrogels is oxidized resulting in amorphous SiO_2 as indicated by FTIR spectroscopy (Figure S4).

Table S2: Summary of mechanical data calculated from unconfined compression measurements by analyzing near-linear regions of stress-strain curves until 10% strain of the gels.

Gel	Compressive modulus (kPa)	Compressive strain at rupture (%)	Compressive strength at rupture (kPa)
1	349.0 (± 20.5)	37.2 (± 3.0)	230.1 (± 43.5)
2	465.5 (± 63.0)	42.2 (± 1.4)	327.6 (± 16.6)
3	254.4 (± 22.9)	36.8 (± 3.3)	150.6 (± 31.4)
4	21.8 (± 4.6)	55.2 (± 5.2)	32.0 (± 3.6)
5	491.8 (± 56)	41.0 (± 0.9)	297.5 (± 55.5)
6	133.2 (± 24.8)	34.8 (± 3.0)	85.1 (± 4.4)
7	24.8 (± 4.6)	35.9 (± 0.6)	14.0 (± 1.9)
8	227.6 (± 64)	39.7 (±1.6)	165.6 (± 15.9)
9	7.4 (± 2.3)	no rupture	[a]1317.0 (± 168.7)
10	3.0 (± 0.3)	no rupture	[a]1031.6 (± 40.9)

[a]For Gels 9 and 10 no rupture was observed in the stress-strain curves until 90 % strain (maximum deformation applied due to security reasons). Therefore, the compressive strength at 90 % strain is reported.

Additional Figures

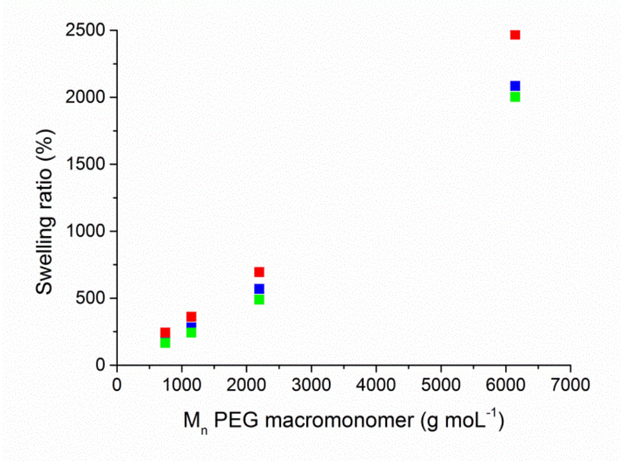

Figure S1. Experimentally found swelling ratios against M_n of PEG macromonomer used to prepare Gels 1-4 at three temperatures (T = 5°C, red squares; T = 25 °C, blue squares; T = 37 °C, green squares). While an almost proportional increase of water uptake for Gels 1-3 is found, a pronounced deviation toward larger swelling ratios is seen for Gel 4. This is in accordance to a pronouncedly decreased gel fraction found for Gel 4 (Table 1).

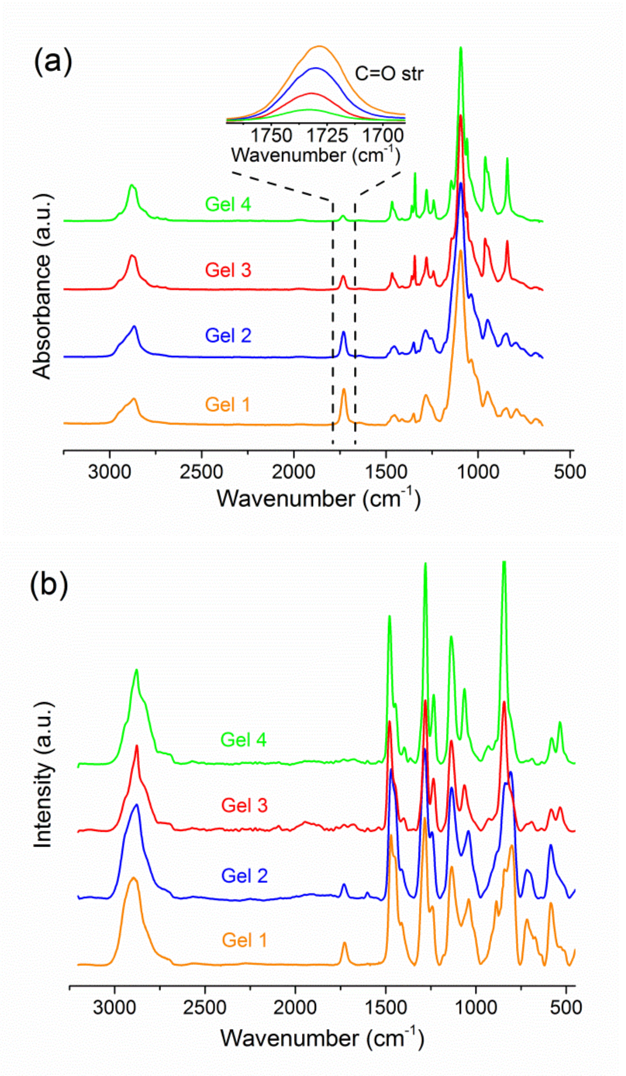

Figure S2. (a) FTIR, and (b) Raman spectra of Gels 1-4. The same spectral features can be observed for Gels 1-4, demonstrating the hydrogels identical linking chemistry. The inset in (a) identifies the gradual reduction of the C=O str band at 1730 cm^{-1} with increased PEG macromonomer chain length. Due to its lower intensity in the Raman spectra it completely vanishes in Gels 3 and 4.

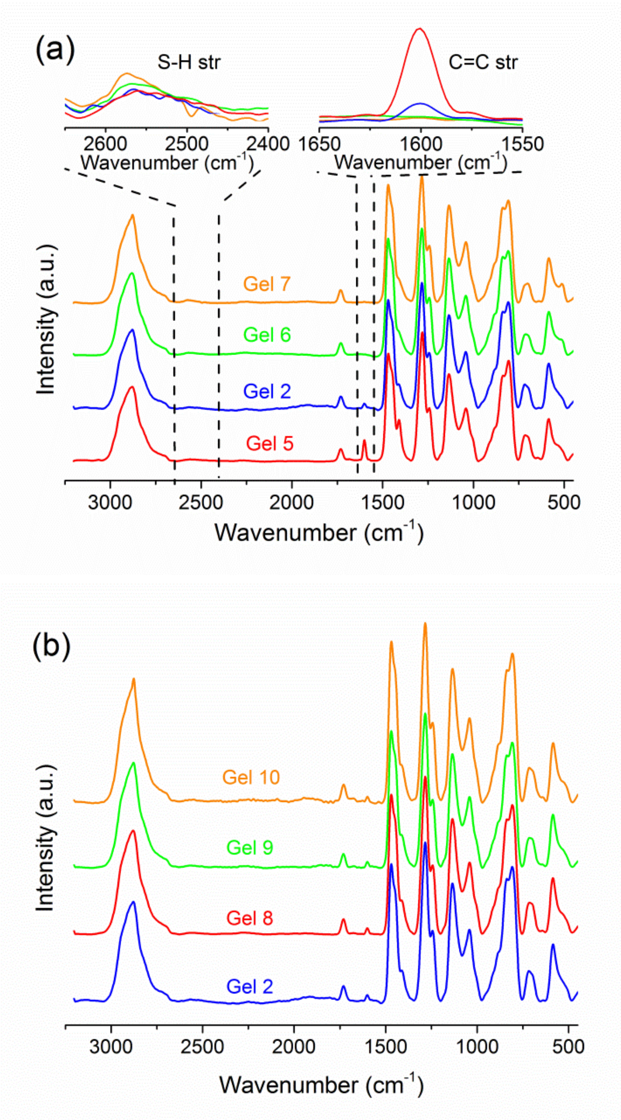

Figure S3. (a) Raman spectra of Gel 2 and Gels 5-7 prepared at increasing thiol:vinyl molar ratios, and (b) Raman spectra of Gel 2 and Gels 8-10 prepared in the presence of different wt% of co-solvent dodecanol. Most evident in (a) is the progressive disappearance of the C=C str at 1600 cm^{-1} accompanied by a progressive increase of the S-H str at 2570 cm^{-1} with the increase of the thiol:vinyl molar ratio. This indicates the step growth nature of the thiol-ene reaction realized here. In (b) one can observe the exactly same spectral features for Gels 2 and Gels 8-10 that reveal very similar intensities for the C=O str band at 1730 cm^{-1} and the very small vinyl str at 1600 cm^{-1}, indicative of the very similar functional group conversions and similar chemical constitution of the gels.

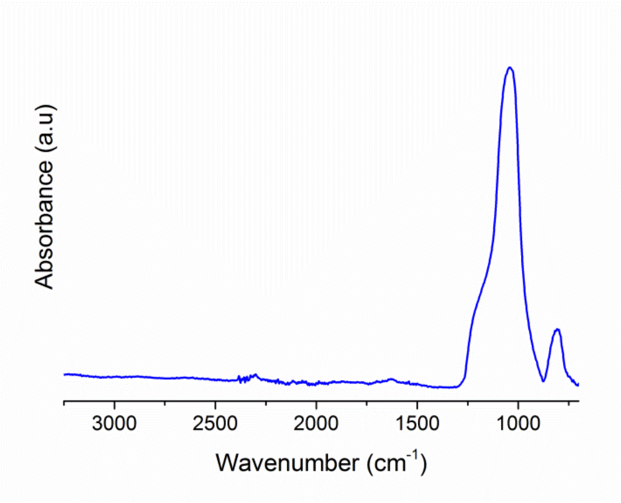

Figure S4. FTIR spectrum of the ceramic yield from TGA of Gel 1. The spectra of the other ceramic yields from the other gels were similar. The observed two bands at 1025 cm^{-1} and 806 cm^{-1} are characteristic of amorphous silica.

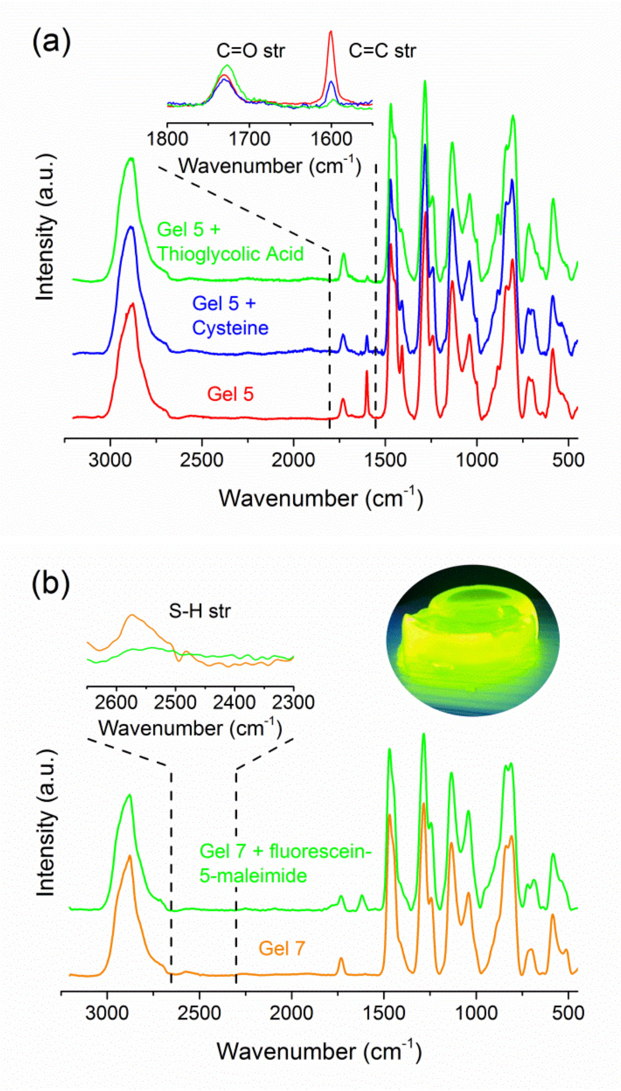

Figure S5. Raman spectra of (a) Gel 5 before (red line), and after functionalization with cysteine (blue line) or thioglycolic acid (green line), and (b) Gel 7 before (orange line) and after functionalization with fluorescein-5-maleimide (green line). In (a) the inset shows the pronounced reduction in intensity of the C=C str band (at 1600 cm^{-1}) in both cases, as well as the increase of the C=O str band (1730 cm^{-1}). The inset in (b) indicates the reduction of the S-H str (at 2570 cm^{-1}) as well as it shows the visual appearance of Gel 7 functionalized with fluorescein-5-maleimide dye under UV-light irradiation (365 nm). The new band at 1619 cm^{-1} can be attributed to the xanthene.

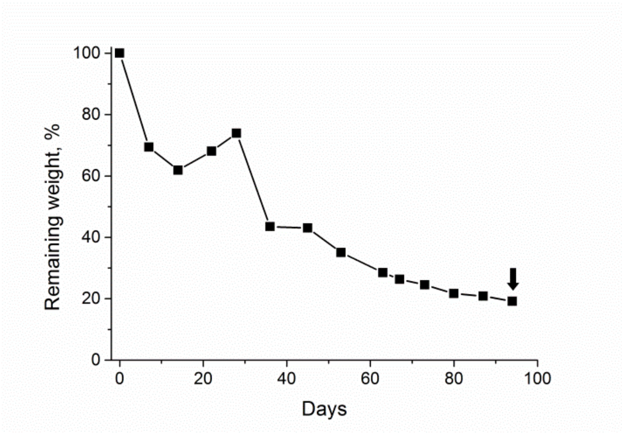

Figure S6. Degradation profile of Gel 1. The arrow indicates remaining weight at degradation time shown in Figure 2b.

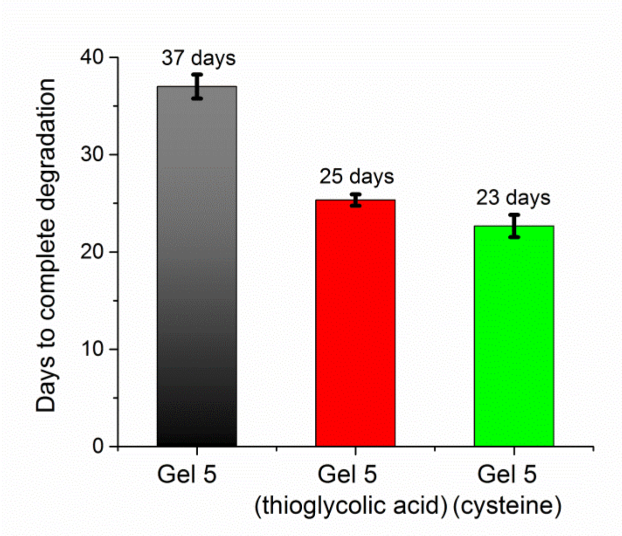

Figure S7. Days to complete degradation for Gel 5 and functionalized variants obtained by thiol-ene addition of thioglycolic acid and cysteine, respectively.

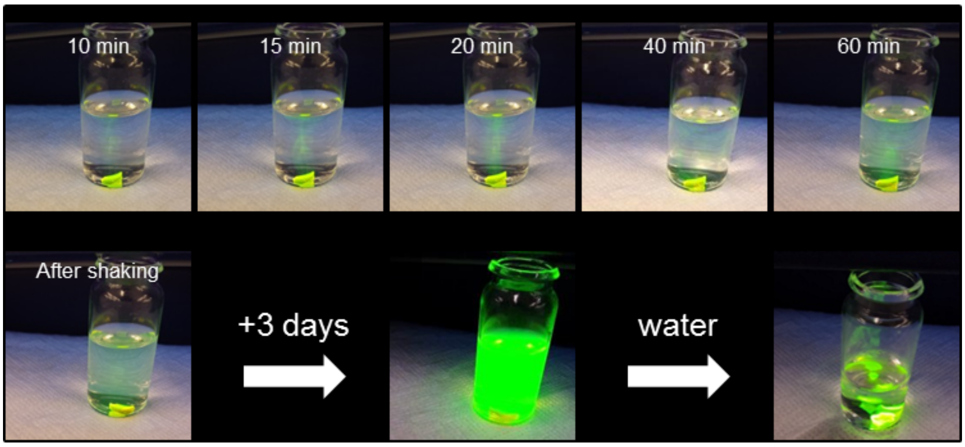

Figure S8. Gel 7, functionalized with fluorescein-5-maleimide, was placed in fridge-cold buffer. After 10 min is clearly seen a release of fluorescein, visually indicative of degradation proceeding with time. After 60 min the gel containing solution was shaken and left for three more days in buffer with the entire solution turning pronouncedly fluorescent. Taking the very soft hydrogel piece and placing it in water showed remaining pieces of hydrogel still being highly fluorescent.

References

1. Y. J. Du and J. L. Brash, *J. Appl. Polym. Sci.*, 2003, 90, 594-607; K. Oberg, Y. Hed, I. Joelsson Rahmn, J. Kelly, P. Lowenhielm and M. Malkoch, *Chem. Commun.*, 2013, 49, 6938-6940.
